# Obstructive sleep apnea in community-dwelling polio survivors: a 5-year longitudinal follow-up study

**DOI:** 10.3389/fneur.2025.1643862

**Published:** 2025-10-10

**Authors:** Qidi Ding, Xiao Li, Meng Wang, Jingyu Wang, Ting Sun, Yunliang Sun, Jianghua Liu, Yan Yu, Jinxian Wu, Juan Du, Xiaosong Dong, Chi Zhang, Yuhua Zuo, Long Zhao, Jing Li, Changjun Lv, Kingman P. Strohl, Fang Han

**Affiliations:** ^1^Department of Sleep Medicine, Peking University People’s Hospital, Beijing, China; ^2^Department of Respiratory and Critical Care Medicine, Binzhou Medical University Hospital, Binzhou, Shandong, China; ^3^Department of the First School of Clinical Medicine, Binzhou Medical University, Binzhou, Shandong, China; ^4^Dongyang People's Hospital, Dongyang, Zhejiang, China; ^5^Dongyang the Seventh People's Hospital, Dongyang, Zhejiang, China; ^6^Division of Pulmonary, Critical Care and Sleep Medicine, Department of Medicine, Case Western Reserve University, and Cleveland Louis Stokes VA Medical Center, Cleveland, OH, United States

**Keywords:** neuromuscular disease, community-living polio survivor, obstructive sleep apnea syndrome, polysomnography, oximetry

## Abstract

**Purpose:**

Obstructive sleep apnea (OSA) is highly prevalent in polio survivors, but longitudinal data on its progression remain limited. This study aimed to characterize OSA progression in community-dwelling polio survivors and compare it with an age-matched control group.

**Methods:**

A prospective 5-year longitudinal study recruited 148 polio survivors (48.76 ± 5.97 years, 75% male). At baseline (2014), all participants underwent overnight oximetry. Among them, 42 completed in-lab polysomnography (PSG) testing. Over the 5-year follow-up, 112 polio survivors (76.79% male, mean age 48.48 ± 6.05 years) were successfully tracked, with 33 undergoing follow-up PSG. Additionally, 59 age- and sex-matched OSA patients were enrolled as controls. Primary outcomes included changes in oxygen desaturation index ≥4% (ODI_4_) and apnea-hypopnea index (AHI). Correlates of OSA progression were analyzed using Pearson’s correlations.

**Results:**

Over 5 years, ODI_4_ increased significantly in polio survivors from 8.11 ± 9.13 to 10.35 ± 11.63 events/h (*p* = 0.01), with a shift toward moderate–severe ODI_4_ (13 to 22%, *p* = 0.027). AHI also rose in both groups: polio survivors (26.57 ± 21.25 to 33.86 ± 22.43 events/h, *p* = 0.02) and controls (27.14 ± 21.91 to 37.24 ± 24.55 events/h, *p* = 0.004), with no significant group difference in AHI progression (*p* = 0.89). However, polio survivors showed increased mixed apnea index (*p* = 0.02) and prolonged REM sleep latency (*p* = 0.009). ODI_4_ changes correlated with scoliosis (*r* = 0.27, *p* = 0.005) and BMI fluctuations (*r* = 0.25, *p* = 0.008).

**Conclusion:**

OSA-related parameters, particularly mixed apnea and REM alterations, progress in polio survivors. Changes in ODI_4_ were positively correlated with BMI fluctuations and scoliosis.

## 1 Introduction

Global estimates suggest 15–20 million polio survivors worldwide, with 2.8 million in China (1). Obstructive sleep apnea (OSA) prevalence among this population ranges from 7.3 to 65% (2), highlighting their substantial demographic presence and the growing public health burden of post-polio sequelae. Multiple mechanistic hypotheses have been proposed to explain OSA pathogenesis in polio survivors, including progressive respiratory muscle dysfunction, neuromuscular scoliosis-induced restrictive lung disease, pharyngeal dilator muscle impairment, upper airway narrowing, or combinatorial effects of these factors (3, 4). However, longitudinal evidence regarding OSA progression in this population remains scarce (5, 6).

Most prior studies on OSA in polio survivors have been cross-sectional and clinically based (7–9). This 5-year longitudinal study aimed to characterize OSA progression in a community-recruited cohort of polio survivors. By comparing outcomes with an age-matched general control group, we sought to determine the role of polio sequelae in OSA progression. To our knowledge, this represents the first longitudinal investigation of OSA in this population. We hypothesized that polio sequelae would be associated with accelerated OSA severity changes over the 5-year follow-up period.

## 2 Methods

This prospective 5-year longitudinal follow-up study was conducted from 2014 to 2019 in Dongyang City, Zhejiang Province, China. The study protocol was approved by the Institutional Review Board of Binzhou Medical University, and written informed consent was obtained from all participants.

### 2.1 Participants

Potential post-polio survivors (defined as individuals with poliovirus exposure >25 years prior) were identified in Dongyang City. We confirmed the history of polio infection through the registration records obtained through collaboration with the local branch of the China Disabled Persons’ Federation, a national agency dedicated to supporting individuals with chronic disabilities. And the time of infection was further determined through systematic interviews. Participants were excluded if they had undergone prior medical treatment for OSA.

### 2.2 Study design and data collection

In 2014, 148 community-dwelling individuals (48.76 ± 5.97 years, 75% male) with a confirmed history of poliomyelitis were recruited. Demographic and clinical data, including age, polio onset age, and sociodemographic factors, were collected via structured interviews. All participants completed the Epworth Sleepiness Scale (ESS) questionnaire. Height and weight were measured using standardized protocols. Medical histories were reviewed to exclude individuals taking sleep-altering medications. Physical examinations, electrocardiograms, chest radiographs, and laboratory tests (complete blood count and metabolic panel) were performed to exclude participants with uncontrolled severe medical conditions.

**Figure 1:**
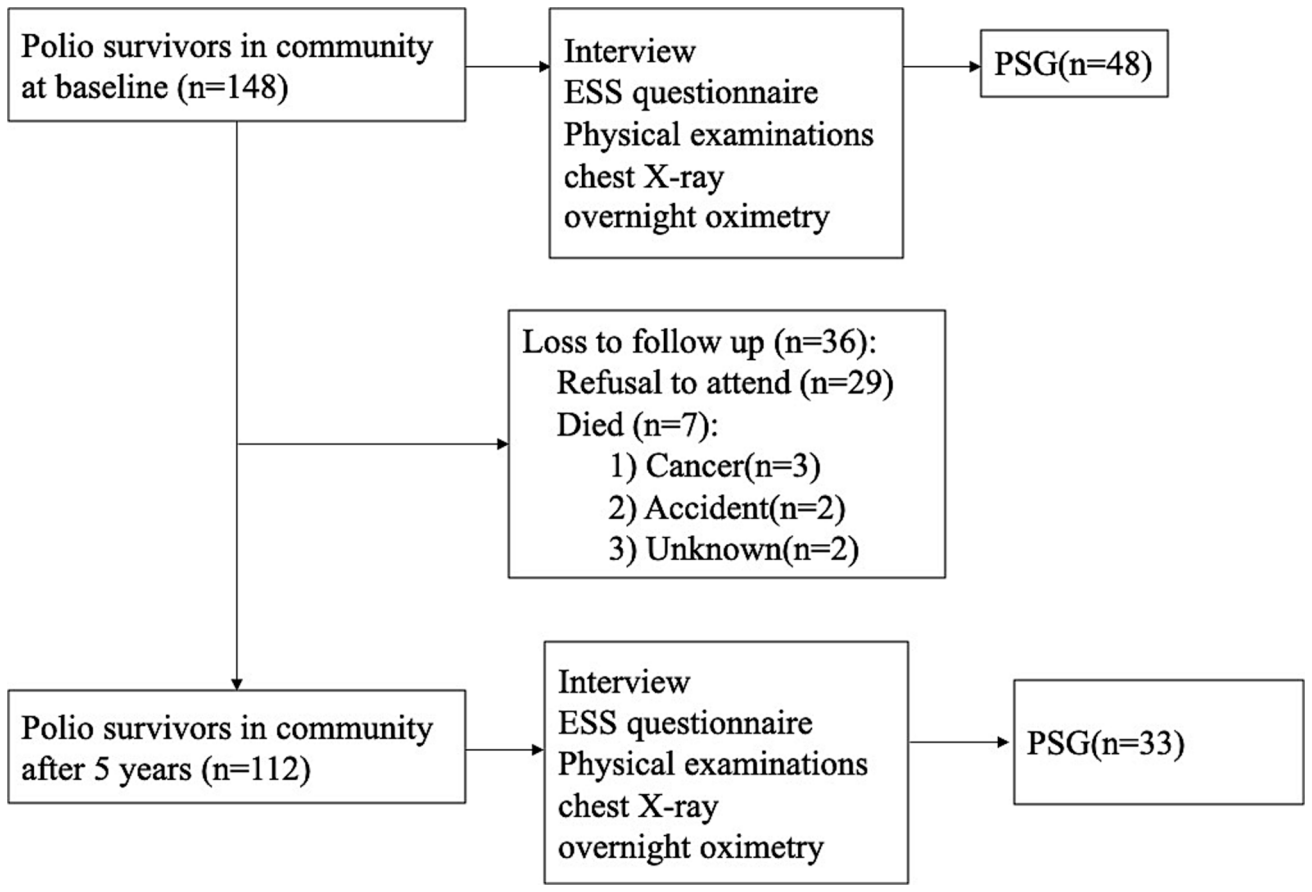
The flow chart of participants. ESS, Epworth Sleepiness Scale; PSG, polysomnography.

As depicted in Figure 1, all 148 participants underwent baseline overnight DS-5 oximetry (KONICA MINOLTA, Osaka, Japan), with 48 completing in-lab standard polysomnography (PSG; Alice series, Philips Respironics, USA). At 5-year follow-up, 112 polio survivors (76%) completed repeat oximetry, 29 (19%) declined participation, and 7 (5%) had died. Of the original PSG cohort, 33 (79%) underwent follow-up PSG (Alice series, Philips Respironics, USA). Sleep stages and respiratory events were scored by trained technicians according to the American Academy of Sleep Medicine (AASM) Scoring Rules, Version 2.3 (10). Apnea was defined as ≥90% airflow reduction for ≥10 s; hypopnea as ≥30% airflow reduction associated with ≥3% oxygen desaturation or arousal. Events were classified as obstructive, central, or mixed per AASM guidelines. The apnea-hypopnea index (AHI) was calculated as the total number of apneas/hypopneas per hour of sleep, with standard cutoffs: normal (<5/h), mild (5–14.9/h), moderate (15–29.9/h), and severe (≥30/h). The oxygen desaturation ≥4% index (ODI_4_) reflected events of ≥4% oxygen desaturation per hour of recording, categorized as normal (<5/h), mild (5–14.9/h), or moderate–severe (≥15/h).

Additionally, at the Sleep Center of Peking University People’s Hospital, we recruited age- and sex-matched OSA patients without prior OSA treatment who had undergone approximately 5 years of follow-up as a general population control group. Demographic data (age, sex), follow-up duration, medical history, and body mass index (BMI) were collected. All controls had completed at least two standard polysomnography (PSG) tests between 1995 and 2019. The control cohort comprised 59 participants (baseline age: 48.15 ± 13 years, 76% male) with a mean follow-up duration of 5.47 ± 1.39 years.

### 2.3 Statistical analysis

Continuous variables are reported as mean ± standard deviation (SD), while categorical variables are presented as counts and percentages. For intragroup comparisons between baseline and follow-up, paired t-tests (parametric data), Mann–Whitney U tests (nonparametric data), and McNemar’s tests (categorical data) were used. Between-group differences for continuous and categorical data were assessed via Student’s t-tests and chi-squared tests (or Fisher’s exact tests for small samples), respectively. To identify factors associated with ODI_4_ changes over follow-up, Pearson’s correlation coefficients were calculated for potential predictors of respiratory parameter changes. Linear multivariate regression analyses were also performed to investigate factors that were associated with the changes in ODI_4_. All statistical analyses were performed using IBM SPSS Statistics 20.0 (IBM, Chicago, IL, USA), with statistical significance defined as *p* < 0.05.

## 3 Results

**Figure 2:**
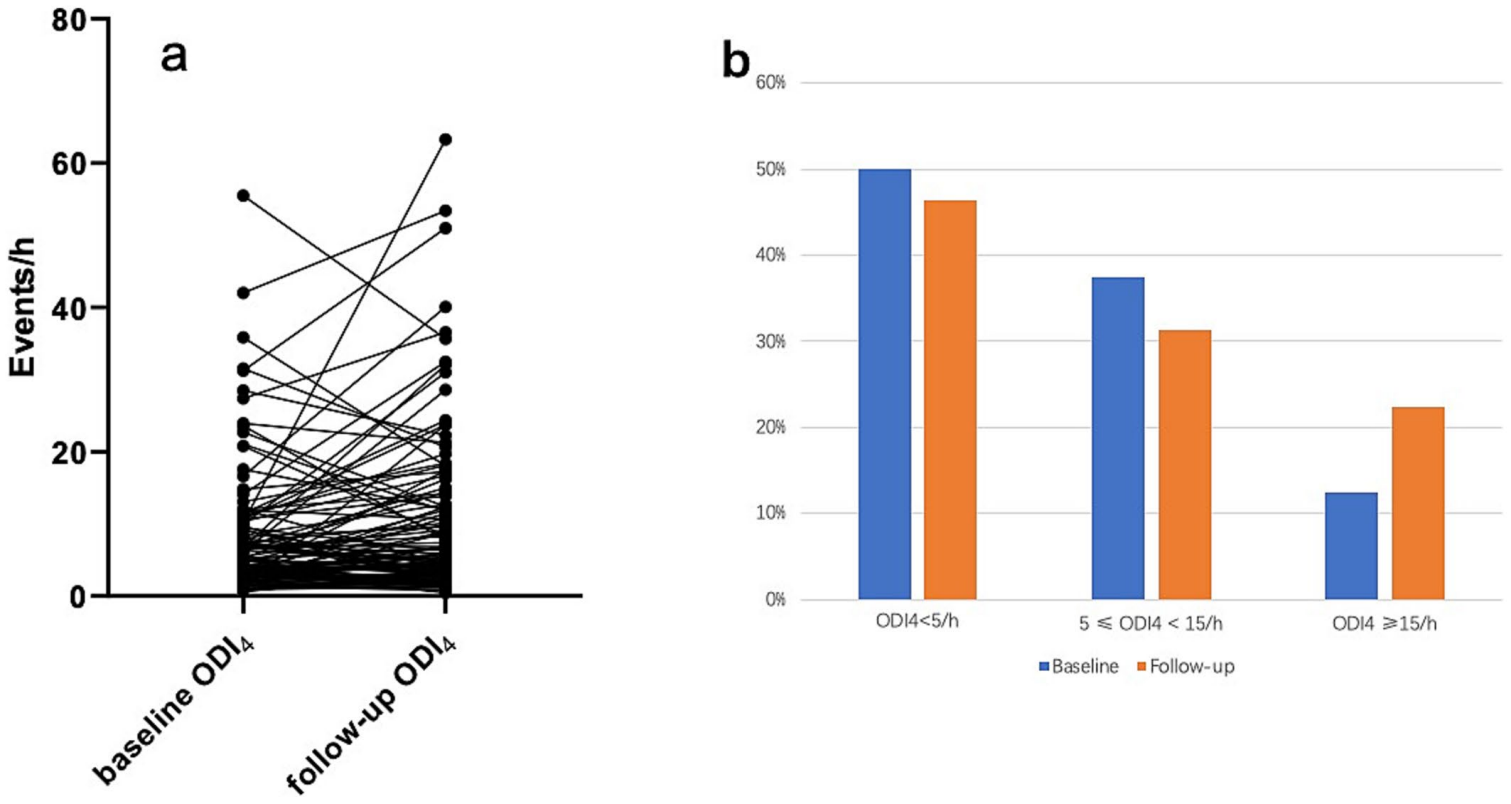
Changes in ODI_4_ of the cohort over 5 years follow-up period. **(a)** Change in ODI_4_ of each polio survivor in the cohort. **(b)** Proportion of participants in the cohort in different ODI_4_ severity group. ODI_4_: oxygen desaturation ≥4% index.

Table 1 presents baseline and follow-up descriptive statistics for all participants and those who completed follow-up. ODI_4_ increased significantly from 8.11 ± 9.13 to 10.35 ± 11.63 events/h (*p* = 0.01) (Figure 2a), with 58% of polio survivors showing ODI_4_ elevation. Baseline ODI_4_ severity distribution (normal: 50%, mild: 38%, moderate–severe: 13%) shifted to 46, 31, and 22% at follow-up (*p* = 0.027) (Figure 2b), reflecting a statistically significant increase in moderate–severe cases.

**Table 1:** Variables for baseline invited participants and actually follow-up cohort.

Variables	Invited participants baseline (*n* = 148)	Follow-up cohort (*n* = 112)	
		Baseline (*n* = 112)	Follow-up (*n* = 112)
Age (year)	48.76 ± 5.97	48.48 ± 6.05	55.53 ± 6.26*
Gender (M%)	75	76.79	76.79
BMI (kg/m^2^)	24.09 ± 3.62	24.30 ± 3.60	24.51 ± 3.55
ESS (points)	6.31 ± 4.29	6.35 ± 4.17	5.35 ± 4.77*
Interval from Polio onset (y)	NA	NA	50.73 ± 6.2
The number of limbs with muscle atrophy	NA	NA	1.38 ± 0.56
Scoliosis (%)	14.86 (22/148)	16.07 (18/112)	16.07 (18/112)
Chest deformity (%)	3.38 (5/148)	3.57 (4/112)	3.57 (4/112)
ODI_4_ (n/h)	7.85 ± 9.95	8.11 ± 9.13	10.35 ± 11.63*
No. (%)			
<5	56.08 (83/148)	50.0 (56/112)	46.42 (52/112)
5 ≤ ~ < 15	32.43 (48/148)	37.5 (42/112)	31.25 (35/112)
≥15	11.49 (17/148)	12.5 (14/112)	22.3 (25/112) *
ODI_3_ (n/h)	10.47 ± 11.29	10.82 ± 10.56	13.85 ± 13.04*
<5	39.9 (59/148)	33.93 (38/112)	25.89 (29//112)
5 ≤ ~ < 15	41.2 (61/148)	45.54 (51/112)	41.96 (47/112)
≥15	18.9 (28/148)	20.54 (23/112)	32.14 (36/112) *
Mean SPO_2_ (%)	95.72 ± 1.74	95.70 ± 1.76	95.08 ± 1.60*
Mini SPO_2_ (%)	79.06 ± 12.85	79.50 ± 11.47	79.14 ± 10.78
Proportion time of SpO_2_ < 90% (%)	2.61 ± 6.02	2.52 ± 5.19	4.12 ± 9.88

BMI, body mass index; ESS, Epworth scale; ODI_4_, oxygen desaturation≥4% index; SPO_2_, pulse oxygen saturation. *p < 0.05.

Change scores (*Δ*) were calculated as follow-up minus baseline values. To identify predictors of ODI_4_ change, correlation analysis evaluated ΔODI_4_ against age, BMI, years since polio onset, scoliosis, and baseline ODI_4_ (Table 2). Significant positive correlations emerged between ΔODI_4_ and scoliosis (*r* = 0.27, *p* = 0.005) and ΔBMI (*r* = 0.25, *p* = 0.008).

**Table 2:** Linear correlation coefficient between baseline clinical, anthropometric, oximeter data and the changes in oximeter data in the follow up periods.

Variables	△ODI_4_ (events/h)	△mean SPO_2_ (%)	△miniSPO_2_ (%)	△Proportion time of SpO_2_ < 90% (%)	△mean pulse (n/min)
Baseline variables					
Age (y)	−0.03	−0.11	0.12	0.31**	0.20*
BMI (kg/m^2^)	0.06	0.07	−0.08	−0.09	0.02
Interval from Polio onset (y)	0	−0.04	0.17	0.026	0.15
Scoliosis	0.27**	−0.11	−0.28**	−0.02	−0.06
ODI_4_(n/h)	−0.19*	0.23*	0.05	−0.06	0.12
Variable changes					
△Age (y)	0.18	0.05	−0.07	−0.39**	−0.12
△BMI (kg/m^2^)	0.25**	−0.12	−0.03	0.10	0.08

BMI, body mass index; ODI_4_, oxygen desaturation≥4% index; SPO_2_, pulse oxygen saturation. △: Variable change value. *p < 0.05, **p < 0.01.

In linear multivariate regression analyses, the change in ODI_4_ was significantly associated with baseline BMI, BMI changes, baseline ODI_4_ and scoliosis (Table 3). While other conditions remain constant, compared with patients without scoliosis, the average change in ODI_4_ in patients with scoliosis is 5.53 n/h higher.

**Table 3:** Linear multivariate regression analyses assessing the association between the change in ODI_4_ and different independent variables.

Variables		Polio survivors	
	*β* (SE)	95%CI	*p* value
Male gender	1.86 (1.86)	[−1.84, 5.56]	0.32
Scoliosis	5.53 (2.21)	[1.14, 9.92]	0.014
Baseline variables			
Age (y)	0.13 (0.14)	[−1.51, 0.40]	0.37
BMI (kg/m^2^)	0.85 (0.28)	[0.31, 1.39]	0.002
ODI_4_ (n/h)	−0.34 (0.11)	[−0.56, −0.12]	0.003
Variable changes			
△Age (y)	0.53 (0.29)	[−0.034, 1.10]	0.07
△BMI (kg/m^2^)	1.11 (0.35)	[0.42, 1.80]	0.002

ODI_4_, oxygen desaturation≥4% index; BMI, body mass index. △: Variable change value.

The 112 survivors who completed follow-up did not differ in age, sex, or BMI from the 33 who underwent PSG follow-up. However, the PSG subgroup had higher baseline ODI_4_ (16.62 ± 13.1 vs. 10.35 ± 11.63 events/h, *p* = 0.009). Table 4 compares baseline and follow-up demographics, anthropometrics, and PSG metrics between polio survivors with PSG follow-up and controls. At baseline, groups were matched for age, sex, BMI, and AHI. The polio group exhibited higher baseline hypopnea index (*p* < 0.001) and greater proportion of respiratory events during REM sleep (*p* = 0.005).

**Table 4:** Demographic, anthropometric and PSG data of the polio survivor cohort and the control group in the baseline and follow-up period.

Variables	Polio survivor (*n* = 33)			Control group (*n* = 59)		
	Baseline	Follow up	*p*	Baseline	Follow up	*p*
Age (years)	50.18 ± 5.28	56.49 ± 5.13	<0.001	48.15 ± 13	53.63 ± 13.12	<0.001
Gender (M%)	87.88 (29/33)	87.88 (29/33)	1	76.27 (45/59)	76.27 (45/59)	1
ESS (points)	8.06 ± 4.87	5.82 ± 5.63	0.05	10.57 ± 4.83	10.48 ± 5.33	0.95
BMI (kg/m^2^)	25.01 ± 5.36	25.27 ± 3.81	0.72	26.7 ± 5.51	26.92 ± 6.0	0.79
Sleep architecture						
TST (min)	399.79 ± 51.75	380.63 ± 55.13	0.15	398.51 ± 86.98	393.24 ± 74.16	0.69
SE (%)	83.41 ± 7.8	84.57 ± 9.57	0.57	87.04 ± 10.66	84.39 ± 13.41	0.19
SL (min)	14.21 ± 11.65	15.72 ± 14.29	0.59	15.96 ± 20.77	12.18 ± 17.39	0.34
REMSL (min)	90.77 ± 41.06*	116.18 ± 51.46	0.002	184.83 ± 104.25	141.33 ± 100.44	0.08
Arousal index (n/h)	19.25 ± 10.25*	28.34 ± 14.77	<0.001	8.7 ± 13.71	28.7 ± 43.14	0.03
N1 (%)	23.07 ± 10.7	21.88 ± 8.08	0.47	26.86 ± 25.16	23.9 ± 20.98	0.51
N2 (%)	42.23 ± 8.89*	44.65 ± 9.78	0.21	56.18 ± 22.03	52.8 ± 20.75	0.46
N3 (%)	17.84 ± 7.91*	17.8 ± 9.05	0.98	4.64 ± 5.31	7.24 ± 10.11	0.05
REM (%)	16.86 ± 4.45*	15.66 ± 4.87	0.25	13.26 ± 9.3	14.78 ± 8.5	0.43
Respiratory events						
AHI (n/h)	26.57 ± 21.25	33.86 ± 22.43	0.02	27.14 ± 21.91	37.24 ± 24.55	<0.001
REM AHI (n/h)	36.44 ± 21.72	40.35 ± 21.97	0.24	28.99 ± 24.8	37.43 ± 24.36	0.08
Proportion of REM event (%)	27.09 ± 17.38*	21.33 ± 13.08	0.05	14.41 ± 13.62	18.23 ± 13.95	0.26
NREM AHI (n/h)	24.78 ± 22.61	31.92 ± 23.37	0.04	27.29 ± 20.28	37.66 ± 25	0.01
AI (n/h)	12.2 ± 19.08	21.03 ± 23.32	0.002	20.32 ± 21.14	30.47 ± 24.59	0.01
OAI (n/h)	9.74 ± 16	10.86 ± 14.19	0.56	17.31 ± 18.1	25 ± 22.7	0.02
MAI (n/h)	1.81 ± 3.82	8.6 ± 11.49	0.001	1.41 ± 3.01	3.28 ± 5.6	0.01
CAI (n/h)	0.65 ± 0.99	1.57 ± 1.93	0.02	0.39 ± 0.92	2.9 ± 7.2	0.02
HI (n/h)	15.14 ± 12.5*	12.83 ± 7.35	0.33	8.93 ± 7.98	8.15 ± 7.33	0.58
Maximum duration of apnea (s)	45.45 ± 20	55.2 ± 23.09	0.001	46.5 ± 17.97	54.76 ± 20.34	<0.01
Proportion of total apnea time (%)	9.42 ± 13.21	16.28 ± 17.68	0.006	14.99 ± 19.89	23.43 ± 21.99	0.005
Maximum hypopnea time (s)	59.75 ± 18.59*	55.57 ± 19.94	0.24	48.26 ± 18.34	52.21 ± 21.82	0.40
Proportion of total hypopnea time (%)	11.07 ± 10.53*	8.83 ± 5.35	0.23	5.65 ± 5.26	5.86 ± 5.46	0.69
ODI_4_ (n/h)	13.15 ± 10.71	16.62 ± 13.1	0.10	13.55 ± 15.95	18.55 ± 17.82	0.03
Mean SpO_2_ (%)	95.33 ± 1.52	94.66 ± 1.45	0.01	95.05 ± 1.41	94.4 ± 2.04	0.05
Lowest SpO_2_ (%)	80.93 ± 9.62	80.93 ± 8.11	0.99	80.21 ± 9.21	77.54 ± 10.08	0.03
SpO_2_ < 90% (%)	3.73 ± 5.17	5.10 ± 7.59	0.25	3.62 ± 5.67	6.78 ± 11.7	0.11
Cardiac events						
Mean heart rate (n/h)	66.98 ± 10.27*	66.47 ± 7.89	0.78	71.93 ± 9.87	69.82 ± 9.41	0.06
Slowest heart rate (n/min)	52.63 ± 6.63*	51.5 ± 4.69	0.35	58.65 ± 14.2	55.94 ± 12.59	0.22
Fastest heart rate (n/min)	90.6 ± 10.21	86.8 ± 7.47	0.04	81.43 ± 21.25	89.4 ± 21.81	0.06

PSG, polysomnography; BMI, body mass index; ESS, Epworth scale; TST, total sleep time; SE, sleep efficiency; SL, sleep latency; REM, rapid eye movement; NREM, non- rapid eye movement; AHI, apnea and hypopnea index; AI, apnea index; OAI, obstructive sleep apnea index; MAI, mixed sleep apnea index; CAI, central sleep apnea index; HI, hypopnea index; ODI_4_, oxygen desaturation ≥4% index; SpO_2_, Pulse oxygen saturation. *p value <0.05 when the baseline data compared to the control group.

**Figure 3:**
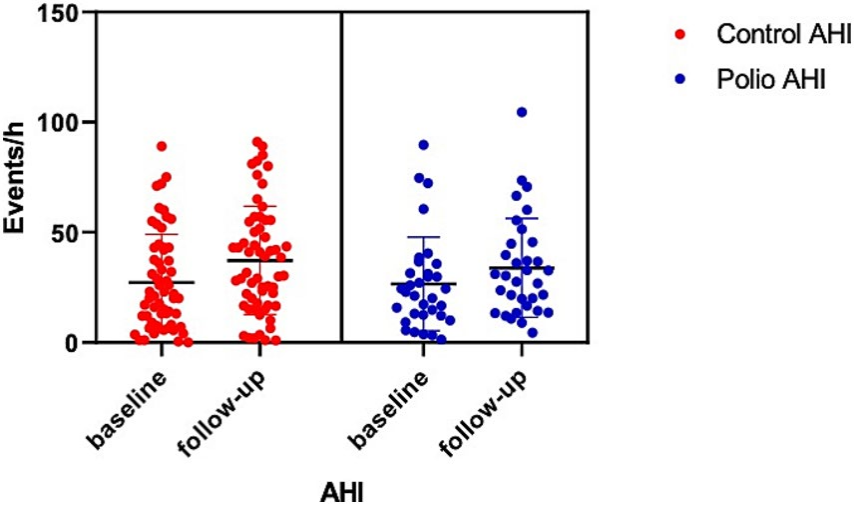
Comparison of AHI between baseline and follow-up in polio survivor group and control group. AHI, apnea and hypopnea index.

In polio survivors, REM sleep latency increased significantly (*p* = 0.002), with a trend toward reduced REM duration. AHI rose significantly in both groups: polio survivors (26.57 ± 21.25 to 33.86 ± 22.43 n/h, *p* = 0.02) and controls (27.14 ± 21.91 to 37.24 ± 24.55 n/h, *p* = 0.004) (Figure 3). Elevated AHI occurred in 73% of polio survivors and 67% of controls. Both groups showed significant increases in maximum apnea duration and total apnea proportion, with slight trends toward reduced SpO₂. Besides, at baseline, the arousal index of polio survivors was already higher than that of the control group. After follow-up, the arousal index in both groups increased significantly.

As shown in Table 5, *Δ*AHI did not differ between groups (*p* = 0.89). However, polio survivors exhibited a significant increase in Δ mixed sleep apnea (MSA, *p* = 0.02) index and ΔREM latency (*p* = 0.009) compared to controls.

**Table 5:** Changes of clinical, anthropometric and PSG data between baseline and follow-up in different groups.

Variables	Post-Polio cohort (*n* = 33)	Control (*n* = 59)	*p*
△Age (y)	5.15 ± 0.87	5.47 ± 1.39	0.18
△BMI (kg/m^2^)	−0.15 ± 2.4	0.22 ± 6.01	0.74
△TST (min)	−19.16 ± 74.68	−5.27 ± 99.85	0.49
△SE (%)	1.16 ± 11.63	−2.65 ± 12.3	0.19
△SL (min)	1.51 ± 15.84	−3.77 ± 25.2	0.3
△REMSL (min)	25.41 ± 44.13	−43.5 ± 147.11	0.009
△N1 (%)	−1.19 ± 8.83	−2.96 ± 28.25	0.71
△N2 (%)	2.43 ± 10.27	−3.38 ± 28.55	0.24
△N3 (%)	−0.04 ± 8.06	2.61 ± 8.23	0.18
△REM (%)	−1.2 ± 5.61	1.51 ± 11.91	0.21
△Arousal index (n/h)	9.08 ± 11.05	20 ± 45.06	0.24
△Maximum duration of apnea (s)	9.75 ± 14.56	8.26 ± 20.17	0.72
△Proportion of total apnea time (%)	6.86 ± 12.7	8.43 ± 20.67	0.71
△Maximum hypopnea time (s)	−4.18 ± 19.19	3.95 ± 29.1	0.20
△Proportion of total hypopnea time (%)	−2.24 ± 10.07	0.21 ± 6.51	0.19
△Total AHI (n/h)	7.29 ± 17.22	10.1 ± 22.1	0.89
△REM AHI (n/h)	3.93 ± 17.75	8.44 ± 30.06	0.73
△NREM AHI (n/h)	7.13 ± 18.34	10.36 ± 24.9	0.72
△AI (n/h)	8.83 ± 14.42	10.16 ± 26.35	0.77
△OAI (n/h)	1.12 ± 10.31	7.69 ± 21.78	0.07
△MAI (n/h)	6.79 ± 10.59	1.87 ± 4.5	0.02
△CAI (n/h)	0.92 ± 2.01	2.51 ± 7.12	0.15
△HI (n/h)	−2.31 ± 12.68	−0.77 ± 9.5	0.55
△ODI_4_(n/h)	3.47 ± 11.75	3.99 ± 15.11	0.87
△Mean SPO_2_ (%)	−0.67 ± 1.48	−0.65 ± 2.15	0.95
△Lowest SPO_2_ (%)	0 ± 8.07	−2.68 ± 8.98	0.18
△SpO_2_ < 90% (%)	1.36 ± 6.58	3.16 ± 11	0.43
△Mean heart rate (n/h)	−0.51 ± 10.25	−2.83 ± 10	0.33
△Slowest heart rate (n/min)	−1.13 ± 6.52	−2.7 ± 16.15	0.53
△Fastest heart rate (n/min)	−3.8 ± 9.84	7.96 ± 29.47	0.01

PSG, polysomnography; BMI, body mass index; TST, total sleep time; SE, sleep efficiency; SL, sleep latency; REM, rapid eye movement; NREM, non-rapid eye movement; AHI, apnea and hypopnea index; AI, apnea index; OAI, obstructive sleep apnea index; MAI, mixed sleep apnea index; CAI, central sleep apnea index; HI, hypopnea index; ODI_4_, oxygen desaturation ≥4% index; SpO_2_, Pulse oxygen saturation; △, Variable change value.

## 4 Discussion

This longitudinal study prospectively evaluated the progression of OSA in patients with polio sequelae using a community-based cohort with a mean follow-up of 5 years. Results demonstrated that ODI_4_, AHI, and maximum apnea duration significantly increased over time in this patient population. Notably, changes in ODI_4_ were positively correlated with changes in BMI and scoliosis.

Several studies have demonstrated that AHI increases progressively over time and that the progression of OSA is correlated with age, male gender, and BMI in the general population (11–13). Mechanistically, older OSA patients have a higher propensity for upper airway collapse than younger ones due to genioglossus neurogenic degeneration and diminished ventilatory sensitivity to oxygen/CO₂ with aging (14–16). Obesity remains the most critical risk factor for OSA progression, as adipose tissue accumulation in the tongue and pharyngeal regions reduces upper airway caliber and enhances collapsibility during sleep (12, 17). Peri-pharyngeal fat deposition additionally contributes to anatomical narrowing of the upper airway (18, 19). However, in aging polio patients, BMI alone may not adequately predict OSA risk due to muscle atrophy-induced weight loss. A shift from muscle to fat mass in these individuals might instead facilitate OSA development (2), highlighting the need for age- and disease-specific risk assessment metrics.

In patients with polio sequelae, residual neuromuscular impairments may promote OSA through multiple mechanisms. Bulbar involvement contributes to hypopharyngeal collapse via hypotonia of upper airway dilator muscles, predisposing patients to obstructive events during sleep (20). Neuromuscular scoliosis and chest wall deformities alter diaphragmatic mechanics, reducing its efficiency in counteracting upper airway collapse (8). Consistent with our findings, results show that changes in ODI_4_ are associated with scoliosis.

Based on the predisposing factors of polio sequelae, we hypothesized that OSA progression in these patients might be more severe than in the general population. However, no statistically significant difference in AHI progression was observed between the polio sequelae group and controls. This may first be attributed to stabilized neurological damage from polio, which does not accelerate its progression. Additionally, our sample comprised community-dwelling patients with relatively mild polio and preserved pulmonary function. We cannot exclude that the follow-up duration was insufficient to detect differences.

Among respiratory events, the dominant types were obstructive apnea and hypopnea, consistent with prior data from Dahan et al. (9) and Hsu et al. (8). At follow-up, the MSA index increased significantly in the polio sequelae group compared to controls. MSA is known to correlate with respiratory control system instability, OSA severity, age, male sex, and obesity (21). Long-term intermittent hypoxia—particularly in severe OSA—may impair respiratory center function through repeated hypoxic stress (22). Thus, the elevated MSA index in this cohort likely reflects worsening OSA. Besides, the high variability in respiratory muscle paresis distribution and bulbar involvement could explain the broad spectrum of SDB pattern observed among polio survivors (4). Further investigation is warranted to clarify these mechanisms.

In the post-polio cohort, we observed a significant increase in REM latency and a trend decrease in REM duration compared to controls, consistent with prior reports of delayed REM sleep onset and reduced REM duration in polio sequelae patients (23). It’s hypothesized that bulbar involvement may indicate additional brainstem poliovirus tropism (particularly in the reticular formation), potentially disrupting REM sleep initiation mechanisms (23). The arousal index was higher in post-polio cohort at baseline. Sliva et al. (24) reported that post-polio patients presented lower sleep efficiency, higher arousal index, higher REM latency and a larger number of stage changes. The increase in the arousal index may be related to factors such as abnormal limb sensations, OSA, and periodic limb movements (PLM). Unfortunately, due to problems like muscle atrophy in this post-polio group, PLM signals could not be accurately identified. Although no significant group difference was found in AHI progression, the elevated proportion of MSA and prolonged REM latency in post-polio patients highlight the characteristics of sleep-disordered breathing compared to controls.

This study has several limitations, including a small sample size, loss to follow-up, and a relatively short follow-up period. Especially a small sample size leads to insufficient statistical power, large sampling errors, poor stability and representativeness of results, making it difficult to detect true differences and limiting extrapolation; it also increases sensitivity to missing data, raises bias risks, and reduces research reliability. Owing to mobility constraints and limited PSG accessibility, the oxygen desaturation index ODI_4_ was employed as a surrogate metric. As reported, when incorporating AHI data from PSG, ODI_4_ demonstrates the highest accuracy in diagnosing and classifying the severity of OSA during nocturnal SpO₂ analysis (25). Although ODI_4_ is less sensitive and specific than the AHI, it reliably tracks longitudinal disease progression. To validate this, we conducted targeted PSG follow-ups where feasible, confirming concordant trends between AHI and ODI_4_ trajectories. Besides, although the sample undergoing PSG follow-up did not differ in age, sex, or BMI from the oximetry group, the PSG subgroup had higher baseline ODI_4_ (16.62 ± 13.1 vs. 10.35 ± 11.63 events/h, *p* = 0.009). There may be selection bias in the sample undergoing PSG follow-up. Because patients with more severe symptoms are more willing to complete in-lab PSG monitoring, the PSG subgroup may fail to accurately reflect the overall changing trends due to more severe baseline OSA. While BMI and scoliosis alone may not fully characterize OSA risk in this population, data limitations—such as the absence of neck circumference measurements and electromyography (EMG) assessments—may have affected the study’s validity. And due to the cross-sectional of the control data, comorbidities such as asthma and diabetes, which can affect the progression of OSA, were not matched. These may also lead to bias in the results.

## 5 Conclusion

This study demonstrates that OSA-related parameters, particularly mixed apnea and REM alterations, progress differently in polio survivors. Changes in ODI_4_ were positively correlated with BMI fluctuations and scoliosis. Despite declining poliomyelitis incidence over recent decades, polio sequelae remain prevalent in aging post-polio populations, posing an escalating challenge for clinical care. Prospective longitudinal follow-up studies with larger samples are needed to inform patient management and optimize treatment strategies.

## Data Availability

The raw data supporting the conclusions of this article will be made available by the authors, without undue reservation.
